# Characterization and Analysis of the Skin Microbiota in Rosacea: Impact of Systemic Antibiotics

**DOI:** 10.3390/jcm9010185

**Published:** 2020-01-09

**Authors:** Yu Ri Woo, Se Hoon Lee, Sang Hyun Cho, Jeong Deuk Lee, Hei Sung Kim

**Affiliations:** 1 Department of Dermatology, Incheon St. Mary’s Hospital, The Catholic University of Korea, Seoul 06591, Korea; w1206@naver.com (Y.R.W.); leesehoon92@gmail.com (S.H.L.); drchos@yahoo.co.kr (S.H.C.); leejd@catholic.ac.kr (J.D.L.); 2 Department of Biomedicine & Health Sciences, The Catholic University of Korea, 222 Banpo-daero, Seocho-gu, Seoul 06591, Korea

**Keywords:** rosacea, papules and pustules, systemic antibiotics, impact, microbiota, microbiome, skin

## Abstract

Systemic antibiotics are extensively used to control the papules and pustules of rosacea. Hence, it is crucial to understand their impact on the rosacea skin microbiota which is thought to be perturbed. The purpose of this study was to compare the makeup and diversity of the skin microbiota in rosacea before and after taking oral antibiotics. We also compared the skin microbiota at baseline according to age and rosacea severity. A longitudinal cohort study was performed on 12 rosacea patients with papules/pustules and no recent use of oral and topical antimicrobials/retinoids. Patients were prescribed oral doxycycline, 100 mg, twice daily for six weeks. Skin areas on the cheek and nose were sampled for 16S ribosomal RNA gene sequencing at baseline, and after six weeks of doxycycline treatment. Eleven females and one male aged 20–79 (median 51) with a median Investigator’s Global Assessment score of 3 (moderate) were enrolled. At baseline, *Staphylococcus epidermidis* was the most dominant species followed by *Cutibacterium acnes* (formerly *Propionibacterium acnes).* In the 60 Over-age group, the prevalence of *Cutibacterium acnes* was lower than that of the 60 & Under-age group. Rosacea severity increased with age and was associated with a decrease in the relative abundance of *Cutibacterium acnes* and an increase of *Snodgrassella alvi.* Across all subjects, antibiotic treatment reduced clinical rosacea grades and was associated with an increase in the relative abundance of *Weissella confusa* (*P =* 0.008, 95% CI 0.13% to 0.61%). Bacterial diversity (alpha diversity) was not significantly altered by antibiotics treatment. Principal coordinates analysis showed mild clustering of samples by patient (ANOSIM, Analysis of Similarity, *R* = 0.119, *P* = 0.16) and scant clustering with treatment (ANOSIM, *R* = 0.002; *P* = 0.5). In conclusion, we believe that rosacea has a unique age-dependent characteristic (i.e., severity). Although we were not able to pinpoint a causative microbiota, our study provides a glimpse into the skin microbiota in rosacea and its modulation by systemic antibiotics.

## 1. Introduction

Rosacea is a chronic, inflammatory skin condition with diagnostic features of persistent facial erythema and phyma. It typically affects the central face. A number of clinical phenotypes such as fixed centrofacial erythema, phymatous changes, papules and pustules, flushing, telangiectasia, ocular manifestations are recognized [1,2]. In terms of pathogenesis, neurovascular dysregulation and aberrant innate immune response are two of the described abnormalities supposed to be involved in rosacea development, both of which can lead to cutaneous inflammation [3]. Evidence that the aberrant innate immune response plays a role in the pathogenesis of rosacea includes upregulation of LL-37 via enhanced processing of cathelicidin by the trypsin-like serine protease kallikrein 5 [4]. When injected to an animal model, cathelicidin peptides induced proinflammatory and angiogenic activity [5], theorizing that dysfunction of the innate immune system causes the clinical features of rosacea (i.e., inflammatory papulopustules).

Patients with rosacea who have papules and pustules are often treated with topical and/or systemic antimicrobials (i.e., metronidazole, ivermectin, erythromycin) [6]. Those with a significant number of papules and pustules require repeated courses of systemic antibiotics which are the same as those applied to inflammatory acne (tetracyclines such as tetracycline, doxycycline, and minocycline). The disappearance of rosacea papules and pustules with systemic antibiotics has been attributed to their anti-inflammatory activity. However, antibiotics (1) leading to complete abolition of the lesions rather than just blunting, and (2) being more effective than agents with much more potent anti-inflammatory effect (i.e., steroids, nonsteroidal anti-inflammatory drugs) suggest that bacteria play a role in the papules and pustules of rosacea.

Microbes such as *Demodex folliculorum, Staphylococcus epidermidis, Bacillus oleronius, Helicobacter pylori*, and *Chlamydia pneumonia* have been addressed to take part in rosacea [7,8,9,10,11], but the results have been inconsistent. As several of the identified microbes are skin commensals, it is difficult to prove that their presence is associated with the disease.

The challenge to characterize the role of microbes in rosacea stems from to the limitations of historic culture-dependent methods in identifying and studying microbes. In addition, rosacea, unlike acne, is distributed across all ages, which makes the interpretation complex as the relative abundance of dominant species varies among different age groups [12].

Amplification and sequencing of the 16S ribosomal RNA (rRNA) gene has been used to determine bacterial communities in various body habitats, including the skin [13]. Using this culture-free method, studies have looked into the action of antibiotics on gut microbiota, often describing changes in bacterial composition following antibiotic treatment [14]. The purpose of this study was to provide an overall picture of the influence of oral antibiotics on the composition and diversity of the rosacea skin microbiota which is supposedly altered. Moreover, we compared the skin microbiota between different age groups (Over 60 vs. 60 & Under) and rosacea severity (Investigator’s global assessment (IGA) score 3 vs. IGA 4).

## 2. Materials and Methods

### 2.1. Study Design

Patients newly diagnosed with rosacea by a dermatologist, were enrolled August 2017–June 2018, at the Department of Dermatology, Incheon St. Mary’s Hospital, Korea. Inclusion criteria were individuals over 18 with more than 10 inflammatory papules/pustules on the face (Grades 3 and 4 on the Investigator’s Global Assessment (IGA) grading scale), and willingness to avoid facial washing and application of topical agents to the face for 12 h prior to skin sampling. Exclusion criteria included history of systemic or topical antibiotic use within one month of the baseline study visit, hypersensitivity to tetracyclines, systemic rosacea treatment within four weeks, topical rosacea treatment within two weeks, significant facial hair interfering with sampling, pregnancy or breast feeding status, and inability to provide an informed consent. The study was approved by Incheon St. Mary’s Hospital (The Catholic University of Korea) Institutional Review Board (OC17TNSI0057), and participants provided written informed consent prior to participation.

### 2.2. Antibiotic Treatment and Sample Collection

Participants were instructed to take doxycycline, 100 mg, twice daily for six weeks. Skin samples were collected at two visits across six weeks, one before treatment initiation and another approximately six weeks after the start of doxycycline therapy. Participants’ compliance to antibiotic treatment was initially checked (by interviewing the patient) at a separate visit made two weeks after the start of doxycycline. Here, the possible side effects of doxycycline such as nausea and diarrhea were inquired. Afterwards, all participants were asked to write a self-reported diary on the medication which was checked six weeks after the starting doxycycline (at the time of visit for the second skin sampling). At each visit (at baseline and after six weeks of doxycycline), skin samples were collected from the cheeks (4 cm^2^ area per side) and nose with a single sterile cotton swab (EASY SWAB, Hanil-Komed Inc., Seongnam, Gyeonggi-do, Korea). All sites (cheeks and nose) were rubbed 20 times with the cotton stick: 10 times in one direction and 10 times perpendicular to this direction. Microbiota sampling was conducted by the same investigator (H.S.K.) at all study visits. 

### 2.3. DNA Extraction and 16S rRNA Gene Polymerase Chain Reaction Amplification and Sequencing

DNA was separated from the skin samples using an enzymatic lysis and bead-based tissue homogenization protocol; the samples were incubated shortly in a lytic enzyme mixture of lysozyme, mutanolysin, proteinase K, and lysostaphin, followed by mechanical lysis with silica beads (0.1 mm), as published beforehand [15]. The DNA cleanup was then conducted with a fecal DNA extraction kit (ZR Fecal DNA MiniPrep; Zymo Research (Irvine, CA, USA). After DNA extraction, the V3–V4 hypervariable region of the 16S rRNA gene was amplified by polymerase chain reaction and sequenced using the Illumina HiSeq platform (250 base pairs, paired-end reads) as reported previously [16,17].

### 2.4. Data Analysis

After sequencing, de-multiplexing of the data based on the Illumina index reads was performed and the raw data were converted to FASTQ files. Illumina adapters were removed using the FASTP program [18] and error-correction was performed on the region where the two reads overlap. The paired reads were merged using FLASH v1.2.11 (http://ccb.jhu.edu/software/FLASH/) [19]. For precise Operational Taxonomic Units (OTUs) analysis, data containing sequence error (i.e., merged sequences shorter than 400 bp, raw reads with ambiguous base cells, chimeric sequences) were removed. The remaining representative reads from non-chimeric clusters were clustered de novo into OTUs (97% similarity threshold) using a CD-HIT-EST-based OTU analysis program (CD-HIT-OTU) (http://weizhongli-lab.org/cd-hit-otu) [20]. Afterwards, taxonomic assignments were performed using the basic local alignment search tool (BLASTN v2.4.0) (blast.ncbi.nlm.nih.gov/Blast.cgi) [21] and the reference database (National Center for Biotechnology Information 16S, Bethesda, MD, USA). Observed relative abundances were estimated by dividing the observed number of 16S rRNA amplicon reads by the total number of reads per sample. Microbiota α diversity, representing microbial diversity within an individual sample, was computed in QIIME v1.9 (http://qiime.org/home_static/dataFiles.html) [22] through the whole tree phylogenetic diversity metric. Microbiota β diversity, which indicates the inter-variability of microbial diversity between samples, was examined through principal coordinates analysis of weighted UniFrac distances in QIIME and hierarchical clustering based on the unweighted pair group method with arithmetic mean algorithm in the R statistical software (r-project.org) (R Core Team). The flexible relationship between the samples were visualized through the PCoA and UPGMA tree. 

### 2.5. Statistical Analysis

Comparison α diversity and relative abundance of bacterial taxa between samples (complete sample sets from pre- and post-treatment groups, sample sets taken before starting treatment based on age (60 & Under vs. Over 60), and rosacea severity (IGA 3 vs. IGA 4)) were performed with Wilcoxon signed rank test/Wilcoxon rank sum test in R package v3.0.1. (cran.r-project.org). All other analyses and visualizations were performed with R and the boxplot package. Permutation tests were used to calculate statistical differences in microbiota in PCoA. For all statistical analyses, two-sided *P* < 0.05 was considered statistically significant.

## 3. Results

Demographics and relevant clinical features of patients included in this study are shown in Table 1 and Table S1. Our study included 12 Asian subjects with skin phototype 3 (50%), and 4 (50%) on the Fitzpatrick scale. One of the subjects (8.3%) was male and the mean age was 49.2 ± 18.6. In terms of rosacea severity (IGA), six patients were IGA grade 3 and six were IGA grade 4 (median IGA: 3) at baseline. After six weeks of oral doxycycline, seven patients were measured to be IGA grade 2 and five patients IGA grade 3 (median IGA: 2). The mean number of papules/pustules was 22.8 ± 9.4 at baseline and 8.6 ± 4.2 after six weeks of doxycycline treatment (*P* < 0.05).

### 3.1. Taxonomic Assignment

**Figure 1:**
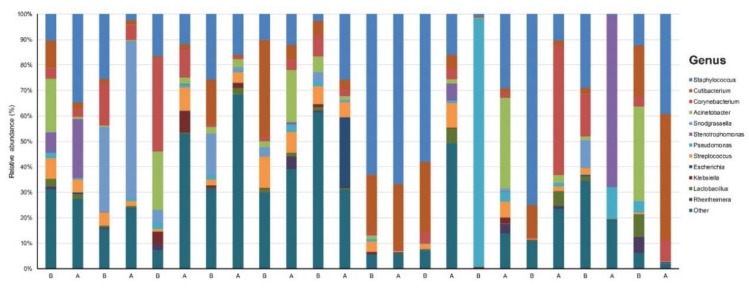
Taxonomy plot of the microbial communities of rosacea patients before (B) and after (A) six weeks of doxycycline.

Our data set involved 24 samples across 12 patients sequenced to a mean (SD) read count of 156,699 (17,769) (Table S2). We identified 16 phyla, 22 classes, 65 orders, 149 families, 390 genera, and 998 species that were unique and present in at least one sample. There was dominance of *Staphylococcus* (24.7%), *Cutibacterium* (10.5%), *Corynebacterium* (7.8%), *Pseudomonas* (5.7%), and *Snodgrassella* (5.6%) at the genus level across all samples (Figure 1).

### 3.2. Relative Abundance of Individual Bacterial Taxa Pre- and Post- Doxycycline Treatment

**Figure 2:**
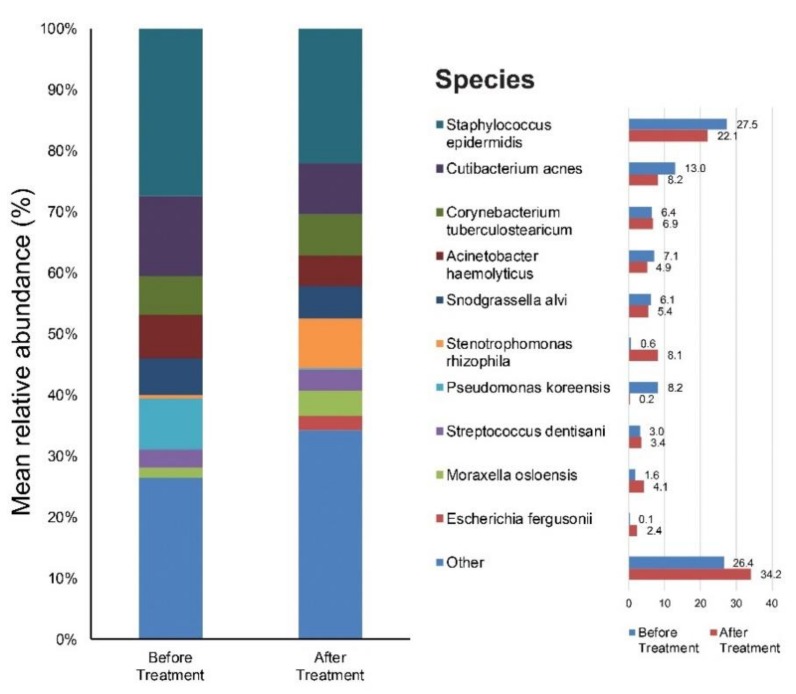
Bar graph on skin microbiota in rosacea patients Before treatment, and after six weeks of doxycycline.

We focused on the species level when assessing changes in abundance of individual bacterial taxa relative to the entire bacterial community in samples. Figure S1A,B provide results of the main bacterial phyla, genus, and species in our 12 subjects at baseline and after six weeks of doxycycline, respectively. *Staphylococcus* (28%), *Cutibacterium* (13%), *Pseudomonas* (9%), *Corynebacterium* (8%), *Acinetobacter* (7%), and *Snodgrassella* (6%) were the main bacterial genera found in untreated rosacea skin. The most dominant taxonomic groups at the species level in rosacea skin were: *Staphylococcus epidermidis* (*S. epidermidis*) (28%), followed by *Cutibacterium acnes (C. acnes)* (13%), *Pseudomonas koreensis* (8%), *Actinetobactor haemolyticus* (7%), and *Snodgrassella alvi (S. alvi)* (6%) (Figure 2). After six weeks of oral antibiotics, the predominant genera were as follows: *Staphylococcus* (22%), *Stenotrophomonas* (33%), *Corynebacterium* (8%) and *Cutibacterium* (7%). Among the bacterial species, *S. epidermidis* (22%) was most commonly found in the skin samples followed by *Stenotrophomonas rhizophila* (8%), *C. acnes* (7%), *Corynebacterium tuberculostearicum* (7%), etc. (Figure 2).

**Figure 3:**
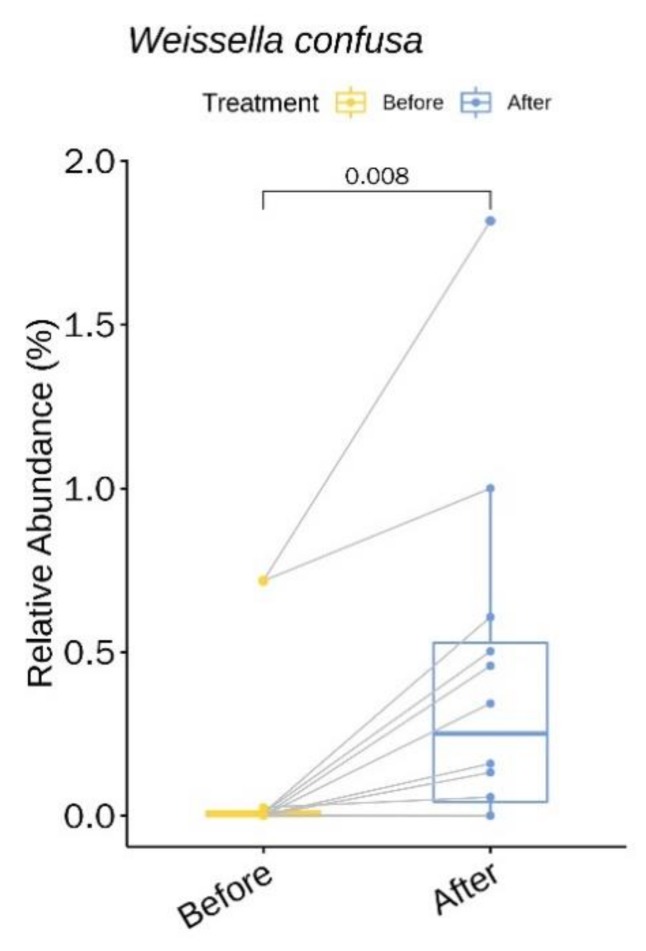
*Weissella confusa* showing a significantly higher relative abundance upon doxycycline treatment.

We identified one genus (with relative abundance of greater than 0.1% across all samples) and one species with statistically significant changes in relative abundance upon treatment with doxycycline (Table S3). *Weissella confusa* increased 3.43-fold (*P* = 0.008, 95% CI 0.13% to 0.61%) following treatment with doxycycline (Figure 3). 

### 3.3. Relative Abundance of Individual Bacterial Taxa at Baseline According to Age, and Rosacea Severity

**Figure 4:**
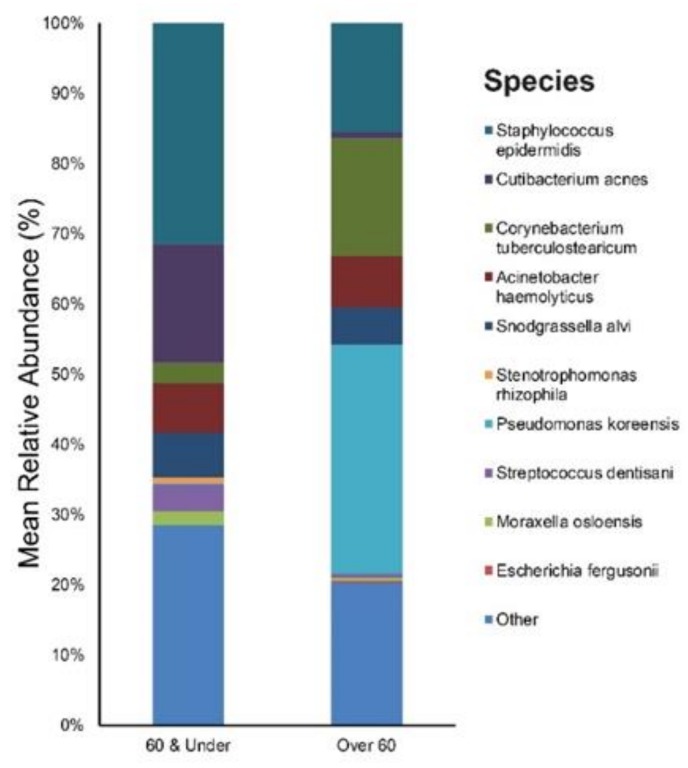
Bar graph on baseline skin microbiota in rosacea patients according to age (60 & Under, and Over 60).

The study participants were divided into two age groups: the young and middle-aged adult group (ages 60 years and under, mean age: 42, n = 9), and the elderly group (ages over 60 years, mean age: 70, n = 3). Figure S2A,B provide results of the main bacterial phyla, genus, and species in our 60 & Under, and Over 60 subjects, respectively. *Staphylococcus* (32%), *Cutibacterium* (18%), and *Snodgrassella* (6%) were the main bacterial genera found in the 60 & Under age group skin. The most dominant taxonomic groups at the species level in 60 & Under skin were: *S. epidermidis* (31%), followed by *C. acnes* (17%), and *S. alvi* (6%) (Figure 4). In the Over 60-age group, the predominant genera were as follows: *Pseudomonas* (33%), *Corynebacterium* (17%) and *Staphylococcus* (16%). Among the bacterial species, *Pseudomonas koreensis* (33%) was most commonly found in the skin samples followed by *Corynebacterium tuberculostearicum* (17%), *S. epidermidis* (10%), *S. alvi* (5%) *etc.* (Figure 4).

**Figure 5:**
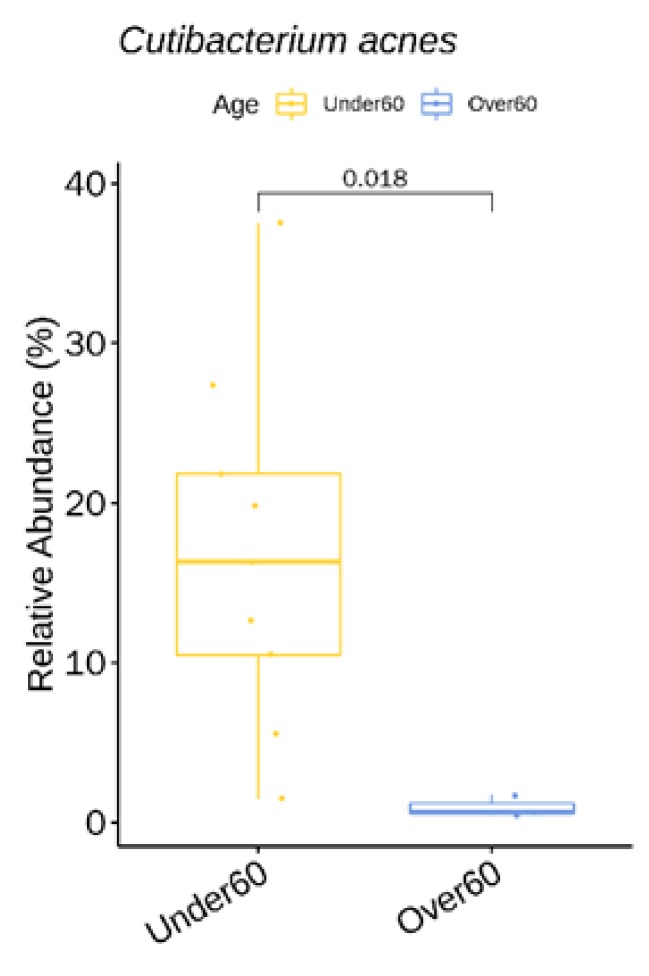
*C. acnes* showing a significantly higher relative abundance in the 60 & Under-age group at baseline.

We identified one genus (with relative abundance greater than 0.1% across all samples) and one species with statistically significant difference in relative abundance between the two age groups (Table S4). *C. acnes* showed a higher relative abundance in the 60 & Under-age group (Figure 5).

**Figure 6:**
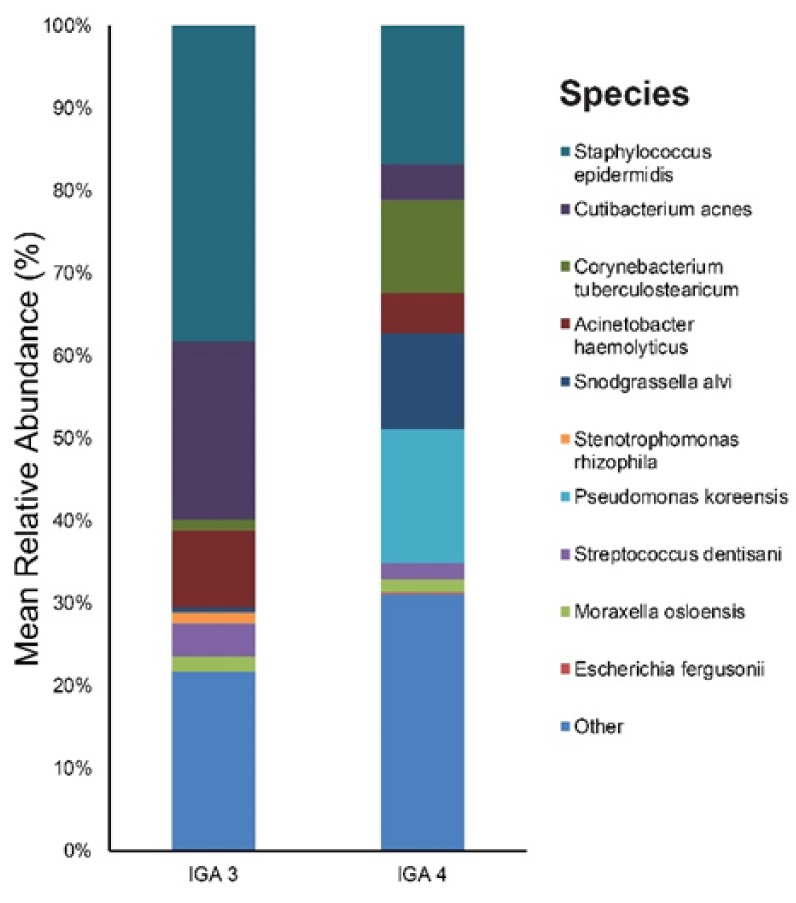
Bar graph on baseline skin microbiota according to rosacea severity (IGA 3 and IGA 4).

Figure S3A,B provides results of the main bacterial phyla, genus, and species according to rosacea severity (IGA 3 and IGA 4) at baseline. *Staphylococcus* (38%), *Cutibacterium* (22%), and *Acinetobacter* (10%), were the main bacterial genera found in the IGA 3 group skin. The most dominant taxonomic groups at the species level in IGA 3 skin were: *S. epidermidis* (38%), followed by *C. acnes* (22%), and *Acinetobacter haemolyticus* (9%) (Figure 6). In the IGA 4 group, the predominant genera were as follows: *Pseudomonas* (18%), *Staphylococcus* (17%), and *Snodgrassella* (12%). Among the bacterial species, *S. epidermidis* (17%) and *Pseudomonas koreensis* (16%) were most commonly found in the skin samples followed by *S. alvi* (12%), *etc.* (Figure 6).

**Figure 7:**
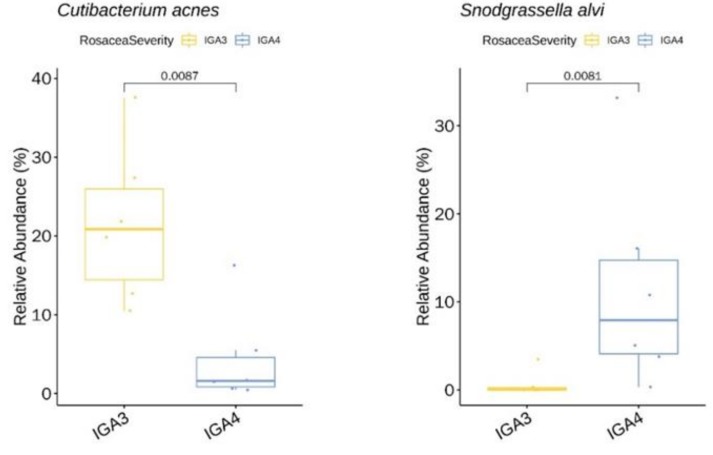
*C. acnes* showing a significantly lower relative abundance in the IGA 4 rosacea severity group at baseline. *Snodgrassella alvi* with a higher relative abundance in the IGA 4 group at baseline.

We identified two genera (with relative abundance greater than 0.1% across all samples) and two species with significant difference in relative abundance between rosacea severity groups IGA 3 and IGA 4 (Table S5). Among these species, *C. acnes* (Figure 7) showed a higher mean relative abundance in IGA 3 skin. In contrast, the relative abundance of *S. alvi* (Figure 7) was higher in the IGA 4 population.

### 3.4. α Diversity

*α* diversity between the before treatment and after treatment group was compared using the inverse Simpson and Shannon indices. *α* diversity over treatment failed to reach statistical significance. Changes in *α* diversity with age (60 & Under vs. Over 60) and rosacea severity (IGA 3 vs. IGA 4) too were not significant.

### 3.5. β Diversity

**Figure 8:**
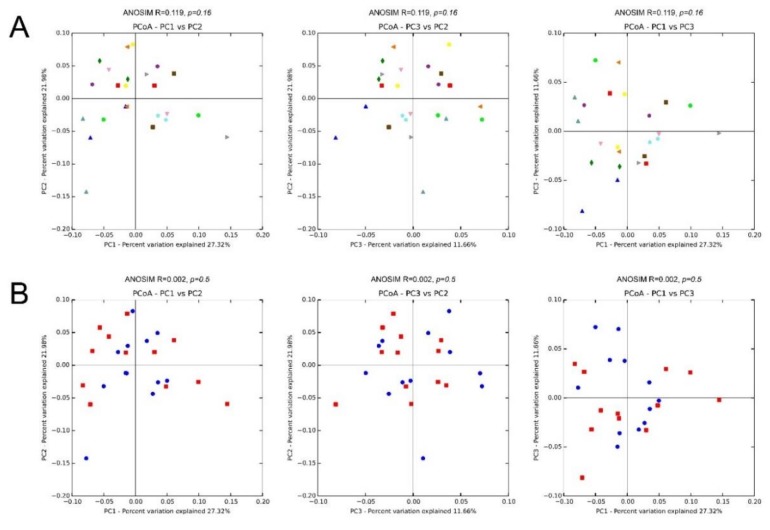
Microbiota β diversity (between-sample microbial diversity) based on principal coordinate analysis (PCoA) of weighted UniFrac distances. Two-dimensional PCoA plots display inter-sample distances by three principal coordinates (PC1, PC2, and PC3) with labeling of individual samples by patient (**A**) and treatment (**B**), for patients 1 to 12. Principal coordinates, calculated from a distance matrix of weighted Unifrac distances and have no units.

We also assessed inter-sample diversity, or *β* diversity, based on principal coordinates analyses of weighted UniFrac distances. Similarity between samples across the three principal coordinates (PC1, PC2, and PC3) with samples that cluster close to one another indicates similar bacterial composition between those samples. The ANOSIM which generates an *R* test statistic stretching from −1 to 1 was used to measure clustering of samples by patient, and treatment (Figure 8). A positive *R* value indicates greater within-group similarity than between-group similarity, with greater magnitudes of the *R* value suggesting stronger clustering of samples. An *R* value of 0 indicates no clustering of samples, whereas a negative *R* value suggests greater between-group resemblance than within-group similarity. There was mild clustering of samples by patient (ANOSIM, *R* = 0.119, *P* = 0.16). Clustering by treatment (ANOSIM, *R* = 0.002; *P* = 0.5) was minimal.

## 4. Discussion

The role of skin microbiota in rosacea is of great interest to clinicians, patients, and researchers. This interest originates from the fact that microbes have long been addressed to take part in rosacea pathogenesis [23,24,25,26] and the efficacy of oral and topical antimicrobials in controlling the papules and pustules of rosacea. Our longitudinal cohort study examined the skin microbiota of untreated patients and the influence of systemic antibiotics on the skin microbiota. Through 16s rRNA gene sequencing, we found *S. epidermidis* and *C. acnes* to be prevalent in the untreated rosacea skin samples, consistent with prior findings [27,28,29,30]. Present in healthy and diseased skin, their roles as commensals or opportunistic organisms are not completely understood. *Corynebacterium kroppenstedtii* was also reported to be abundant in patients with rosacea [30] but was surprisingly scarce in our study population.

Cyclines are broad-spectrum bacteriostatic antibiotics and are commonly prescribed for those who have significant number of papules and pustules [31]. Tetracyclines have multiple mechanisms of action including antibacterial effect, inhibition of pro-inflammatory mediators and tissue destructive enzymes, and modulation of innate immunity, but it is not known which mechanism is the most relevant in attenuating the papules/pustules. As for our study, treatment with doxycycline clearly led to a decrease in rosacea severity (IGA) and the number of inflammatory papules/pustules.

Our findings of significant changes in relative abundance (%) of *Weissella confusa* may have important clinical implications. As for the bacteria, we observed a 3.43-fold increase after six weeks of doxycycline treatment. *Weissella spp.* are Gram-positive, catalase-negative, alpha hemolytic bacteria that appear as short rods or coccobacilli in pairs and chains [32]. Based on their unusual Gram stain morphology, they have often been confused with *Lactobacillus spp.* or *viridans* streptococci [33]. *Weissella* is a common inhabitant of the gut flora but not a part of the normal skin flora [33]. The clinical significance of *Weissella confusa* remains unclear in the setting of polymicrobial infections. *Weissella confusa* is found in fermented foods and has been suggested as a probiotic [34]. In particular, the cell-free culture supernatant of *Weissella confusa* carries various beneficial characteristics such as antibacterial potential and anti-inflammatory efficiency [35]. However, one should carefully interpret its increase as *Weissella confusa* has also been associated with sepsis and other serious infections in humans [36]. In fact, the abundance of *Weissella* genera showed positive correlation with rosacea severity in a twin study [27] (univariate random effect Poisson regression), which contradicts our findings.

Prior studies on skin disease (i.e., atopic dermatitis and inflammatory acne) have shown less bacterial diversity in the disease state [37] which was restored after systemic antimicrobial treatment [38,39]. As for our study, the *α* diversity (within-sample microbial diversity) failed to reach statistical significance over treatment. This may be due to the small number of our study participants (n = 12), but also suggests that an altered collective skin microbiota rather than a single culprit is involved in rosacea pathogenesis.

As for *β Diversity*, which is the inter-sample diversity, there was mild clustering of samples by patient (ANOSIM, *R* = 0.119; *P* = 0.16), indicating a distinctive microbial signature for each person. On the other hand, clustering by treatment (ANOSIM, *R* = 0.002; *P* = 0.5) was minimal, which is consistent with the knowledge that the skin microbiota is highly individualized with specific bacterial taxa persisting on a given person for months to years. The finding emphasizes the importance of taking paired samples from the same patient.

Age is known to influence skin microbiota composition, which partially explains the difference in microbial pathogenesis (*C. acnes* involvement) between acne and the papules and pustules of rosacea. As for our rosacea study population (mean age 49.2 ± 18.6), *S. epidermidis* (28%) was most prevalent followed by *C. acnes* (13%). This strikingly differs from our acne population data (n = 20, mean age 19.6 ± 7.5) where the most dominant taxonomic groups at the species level were: *C. acnes* (25%) followed by *S. epidermidis* (19%) (unpublished data). The relative abundance of *C. acnes* in our Over 60 age group (mean age: 70) was lower than that of the 60 & Under age group (mean age: 42) (0.9% vs. 17%) which is compatible with prior study results. Bensaleh et al. have associated changes in hormonal levels during menopause to the decrease of *Cutibacterium* species observed in the older age group [40]. Zhai et al. Ref. [12] have reported that *C. acnes* evolves with age, showing the lowest abundance in childhood (four–six years) (2.1%), a dramatic increase at puberty (11–13 years) (13.5%), a peak in young adults (25–34 years) (40.3%) and a subsequent decline with age (Middle age adults (37–53 years) (27.2%), and elderly (62–74 years) (8.7%)). It is notable that there is a sharp decline in *C. acnes* after the age 60. The elderly population were also reported to have more bacterial diversity than the other age groups [12,41], but we could not confirm this due to the small number of study participants.

Sex is also thought to influence the composition and diversity of the bacterial communities but to a lesser extent compared to age [12]. We were unable to personally look into this because of our predominantly female study population (11 females vs. one male). Still, we found a high relative abundance of *Pseudomonas* (33%) in the Over 60-age group at baseline, which is in line with findings from a prior study where elderly females had a greater abundance of *Pseudomonas, Kocuria,* and *Flavobacterium* than their male counterparts [12]. This variation is thought to rise from the difference in sebum level as well as the habitual use of cosmetics and moisturizers among the female population.

As for rosacea severity, disease severity was associated with higher age (mean age of the IGA 3 group: 36 vs. mean age of the IGA 4: 63 years, *P* < 0.05). Unlike acne, rosacea usually progresses over time. The age-dependent severity of rosacea has also been reported by Zaidi et al. [27] where rosacea was found to be more severe in the 30–60 age group compared to the 0–30 group. Although rosacea can develop in many ways and at any age, patient surveys indicate that it typically begins any time after age 30 as flushing or redness on cheeks, nose, chin or forehead that come and go. There is progression of the disease with time which likely explains the higher severity of rosacea in older ages. The prevalence of *C. acnes* and *S. alvi* was associated with the severity of rosacea (*C. acnes*: negative association and *S. alvi*: positive association). *S. alvi,* a species known as gut symbiont of bees has been identified as a core microbiota of *Demodex* mites from rosacea patients [24] and was also prevalent (6%) in our untreated skin samples. Interestingly, *S. alvi* has also been associated with inflammatory acne, with a significant decrease in its relative abundance after six weeks of doxycycline treatment (our study, data not shown). Although our findings suggest a subtle link between rosacea and *demodex* infestation, there is some doubt on the origin of *S. alvi.* Since *Demodex mites* usually reside in the hair follicles and sebaceous glands, they would not have been thoroughly collected through skin swabbing.

A negative correlation between *C. acnes* and rosacea severity was also observed by Rainer et al. [30], and though we underrated its significance, the protective role of *C. acnes* in maintaining healthy skin [42] should be appreciated. *C. acnes* can prevent other microbes from colonizing the skin by breaking down sebum into free fatty acids [43]. The lack of *C. acnes* in patients with rosacea [44] has been suggested as evidence that *C. acnes* does not play a major role in the pathogenesis of rosacea, but perhaps it is the deficiency of *C. acnes* compared to the healthy (or younger) population that is significant. *C. acnes* abundance has, in fact, been found to be decreased in certain skin diseases such as atopic dermatitis and psoriasis [45,46].

A potential limitation of our study is the low sample size. Moreover, the use of swabs for skin sampling may have failed to capture the bacterial community of the pilosebaceous unit. Although a recent study by Hall et al. [47] showed no difference in *C. acnes*-associated factors between surface and follicular sampling methods, simultaneous examination of the follicular microbiota may add insights on shifts in the skin microbiota observed in the present study.

Our study did not include further follow-up, but it would be interesting to explore what happens to the skin microbiota in rosacea after completion of the treatment course, and how quickly the bacterial communities return to their initial state, may this happen.

Finally, we were unable to obtain strain-level resolution of the bacteria. As the pathogenicity of different strains within the same bacterial species can vary, it would be valuable to identify the bacterial strains in rosacea and examine their alteration with antibiotic treatment.

## 5. Conclusions

We find rosacea to have a unique age-dependent characteristic (i.e., severity). Although we were not able to pinpoint a causative microbiota, our study provides a glimpse into the skin microbiota in rosacea and its modulation by systemic antibiotics.

## Figures and Tables

**Table 1:** Demographic and clinical characteristics of the study participants.

General Characteristics (n = 12)	
Sex (M/F), n (%)	1/11 (8.3%)
Age (years), median (range)	51 (20–79)
Fitzpatrick Skin Type, median (range)	3 (3–4)
Duration of Rosacea (years), median (range)	2 (less than a year–10)
Baseline Rosacea severity (IGA), median (range)	3 (3–4)
Rosacea severity (IGA) after 6 weeks of oral doxycycline, median (range)	2 (2–3)

## Supplementary Materials

The following are available online at https://www.mdpi.com/2077-0383/9/1/185/s1, Figure S1: Krona graph on skin microbiota in rosacea patients. Figure S2: Krona graph on baseline skin microbiota in rosacea patients (A) 60 & Under, and (B) Over 60 years of age. Figure S3: Krona graph on baseline skin microbiota according to rosacea severity. Table S1: title, Demographic and Clinical Characteristics of the Study Participants in detail. Table S2: Sample read counts. Table S3: Bacterial genera (with relative abundance of greater than 0.1% across all samples) and species with significant changes in mean relative abundance after doxycycline treatment. *Weissella* showed a higher relative abundance following treatment. Table S4: Bacterial genera (with relative abundance of greater than 0.1% across all samples) and species with significant difference in relative abundance between the two age groups (60 & Under, Over 60) at baseline. *Cutibacterium* showed a higher relative abundance in the 60 & Under-age group. Table S5: Bacterial genera (with relative abundance of greater than 0.1% across all samples) and species with significant difference in relative abundance between rosacea severity (IGA) 3 and IGA 4 group at baseline.
