# Supplementary material for: Characterization and Analysis of the Skin Microbiota in Rosacea: Impact of Systemic Antibiotics
Source: J Clin Med. 2020 Jan 9;9(1):185. doi: 10.3390/jcm9010185 (PMC7019287; doi:10.3390/jcm9010185)
Supplement: Supplementary file 1 [file jcm-09-00185-s001.pdf]

Supplementary File

Characterization and Analysis of the Skin Microbiota in Rosacea: Impact of Systemic Antibiotics

Yu Ri Woo [1], Se Hoon Lee [1], Sang Hyun Cho [1], Jeong Deuk Lee [1] and Hei Sung Kim [1,2,*]

[1] Department of Dermatology, Incheon St. Mary's Hospital, The Catholic University of Korea, Seoul 06591, Korea; w1206@naver.com (Y.R.W.); leesehoon92@gmail.com (S.H.L.); drchos@yahoo.co.kr (S.H.C.); leejd@catholic.ac.kr (J.D.L.)

[2] Department of Biomedicine & Health Sciences, The Catholic University of Korea, 222 Banpo-daero, Seocho-gu, Seoul 06591, Korea

* Correspondence: hazelkimhoho@gmail.com; Tel.: +82-32-280-5105

Table S1. Demographic and Clinical Characteristics of the Study Participants in detail.

No.	Sex	Age	Race	IGA Score (Before)	IGA Score (After)	No. of Papules (Before)	No. of Papules (After)	Prior Rosacea Treatment at Baseline	Use of Lotions and Makeup*
1	F	46	Asian	3	2	11	4	None	None/None
2	F	60	Asian	4	3	31	12	None	None/Lotion
3	F	66	Asian	4	3	33	15	None	None/None
4	F	55	Asian	4	3	32	11	None	None/None
5	F	51	Asian	3	2	12	4	None	None/None
6	F	51	Asian	4	3	28	16	None	Lotion, Makeup/None
7	M	20	Asian	3	2	16	8	None	None/None
8	F	49	Asian	3	2	17	5	None	None/None
9	F	66	Asian	4	3	25	12	None	None/None
10	F	23	Asian	3	2	13	5	None	Lotion/None
11	F	79	Asian	4	2	40	5	Oral doxy last taken 4 months ago	None/None
12	F	24	Asian	3	2	16	6	None	None/None

* Use of lotion and makeup on the skin the day of sampling (baseline, after doxycycline) noted by subject.

Table S2. Sample read counts.

Sample No	Before/After Doxycycline	Read Count (CD-HIT-OUT)
1	Before	141,709
	After	168,785
2	Before	156,669
	After	145,979
3	Before	152,790
	After	180,183
4	Before	164,650
	After	164,531
5	Before	148,613
	After	186,180
6	Before	202,724
	After	158,722
7	Before	179,044
	After	169,249
8	Before	167,400
	After	148,913
9	Before	156,140
	After	154,513
10	Before	143,796
	After	152,680
11	Before	138,044
	After	149,772
12	Before	132,954
	After	119,261

Table S3. Bacterial genera (with relative abundance of greater than 0.1% across all samples) and species with significant changes in mean relative abundance after doxycycline treatment. *Weissella* showed a higher relative abundance following treatment.

Genera	Fold-Increase	*p*-Value	95% CI
Weissella	3.43	0.008	0.13–0.61%
**Species**	**Fold-Increase**	***p*-Value**	**95% CI**
Weissella confusa	3.43	0.008	0.13–0.61%

Table S4. Bacterial genera (with relative abundance of greater than 0.1% across all samples) and species with significant difference in relative abundance between the two age groups (60 & Under, Over 60) at baseline. *Cutibacterium* showed a higher relative abundance in the 60 & Under-age group.

Genera	Fold-Increase	*p*-Value	95% CI
Cutibacterium	14	0.02	1.18–36%
**Species**	**Fold-Increase**	***p*-Value**	**95% CI**
Cutibacterium acnes	18	0.02	0.99–36%

Table S5. Bacterial genera (with relative abundance of greater than 0.1% across all samples) and species with significant difference in relative abundance between rosacea severity (IGA) 3 and IGA 4 group at baseline. Those with significantly higher mean relative abundance in the (**A**) IGA 3, and (**B**) IGA 4 group.

(**A**)

Genera	Fold-Increase	*p*-Value	95% CI
Cutibacterium	4.68	0.009	7.08–27%
**Species**	**Fold-Increase**	***p*-Value**	**95% CI**
Cutibacterium acnes	4.97	0.009	7.14–27%

(**B**)

Genera	Fold-Increase	*p*-Value	95% CI
Snodgrassella	18	0.008	0.34–30%
**Species**	**Fold-Increase**	***p*-Value**	**95% CI**
Snodgrassella alvi	18	0.008	0.34–30%

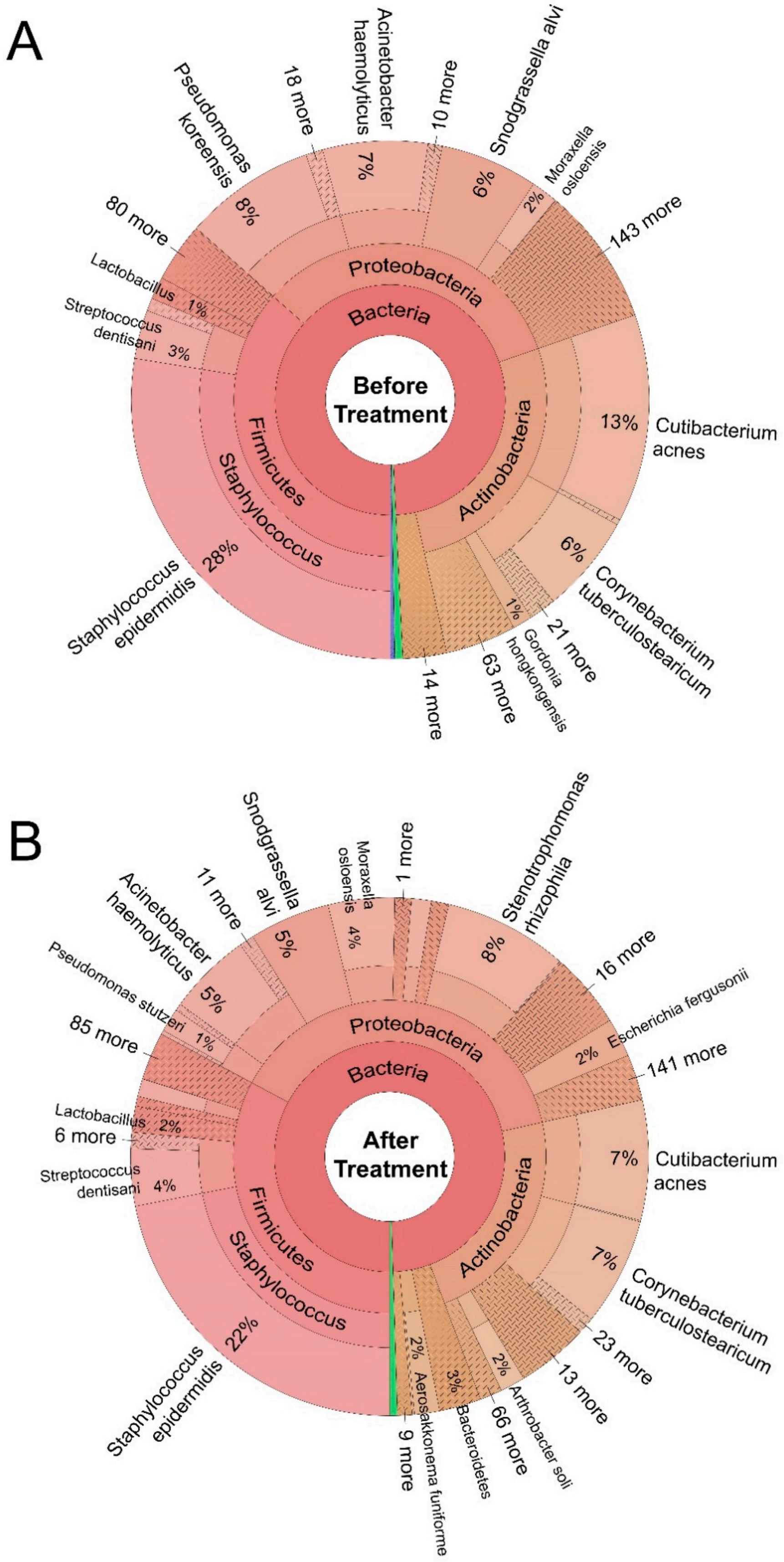

Figure S1. Krona graph on skin microbiota in rosacea patients (**A**) Before treatment, and (**B**) After 6 weeks of doxycycline.

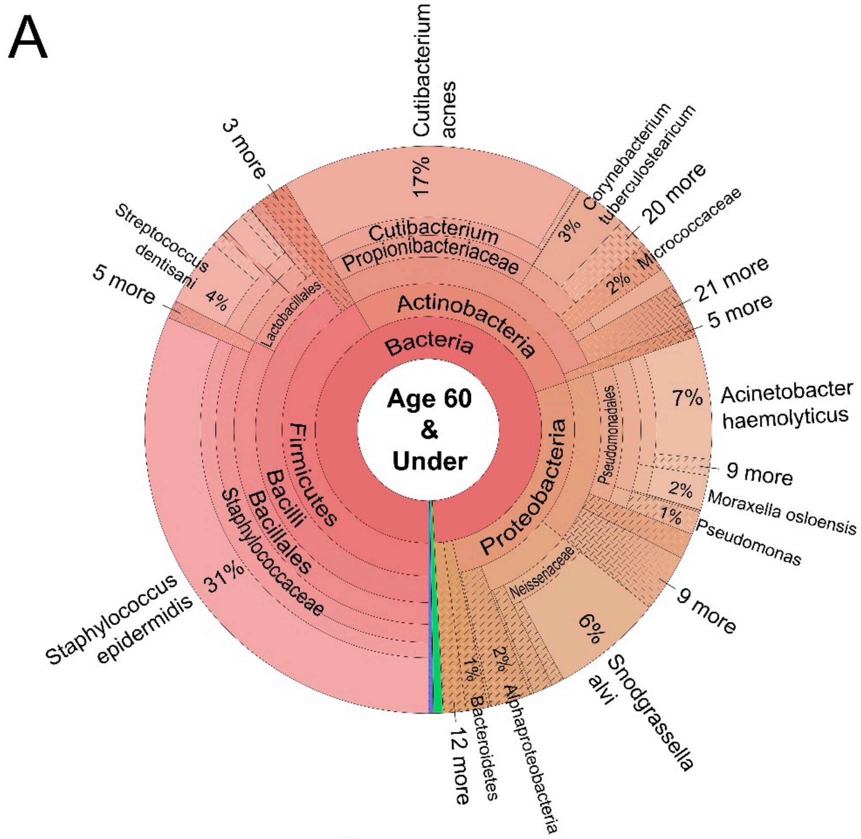

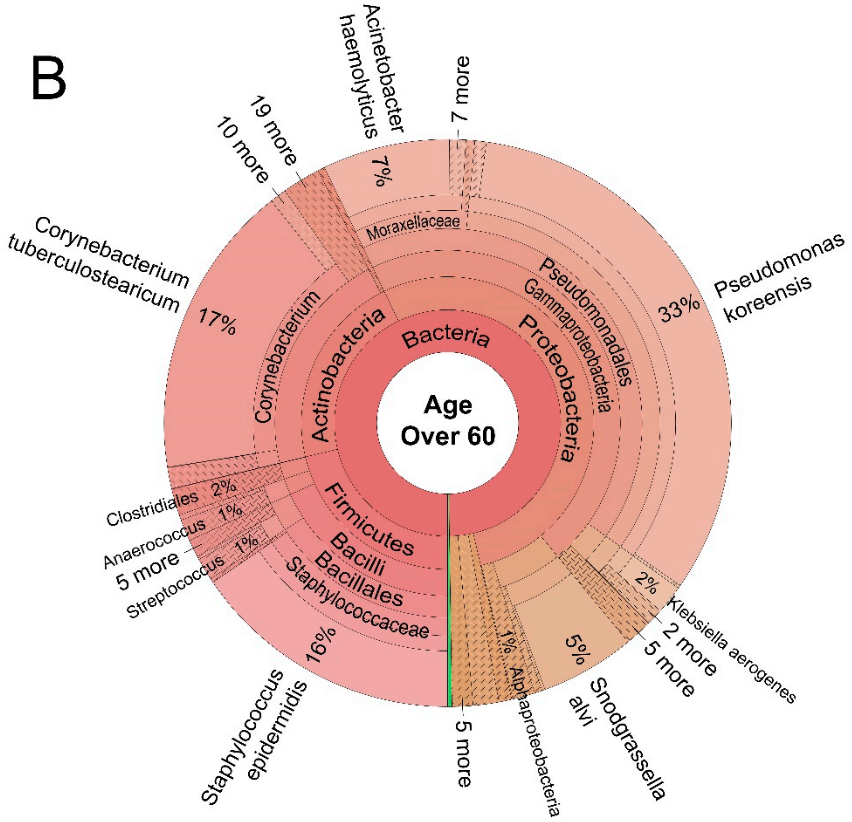

Figure S2. Krona graph on baseline skin microbiota in rosacea patients (**A**) 60 & Under, and (**B**) Over 60 years of age.

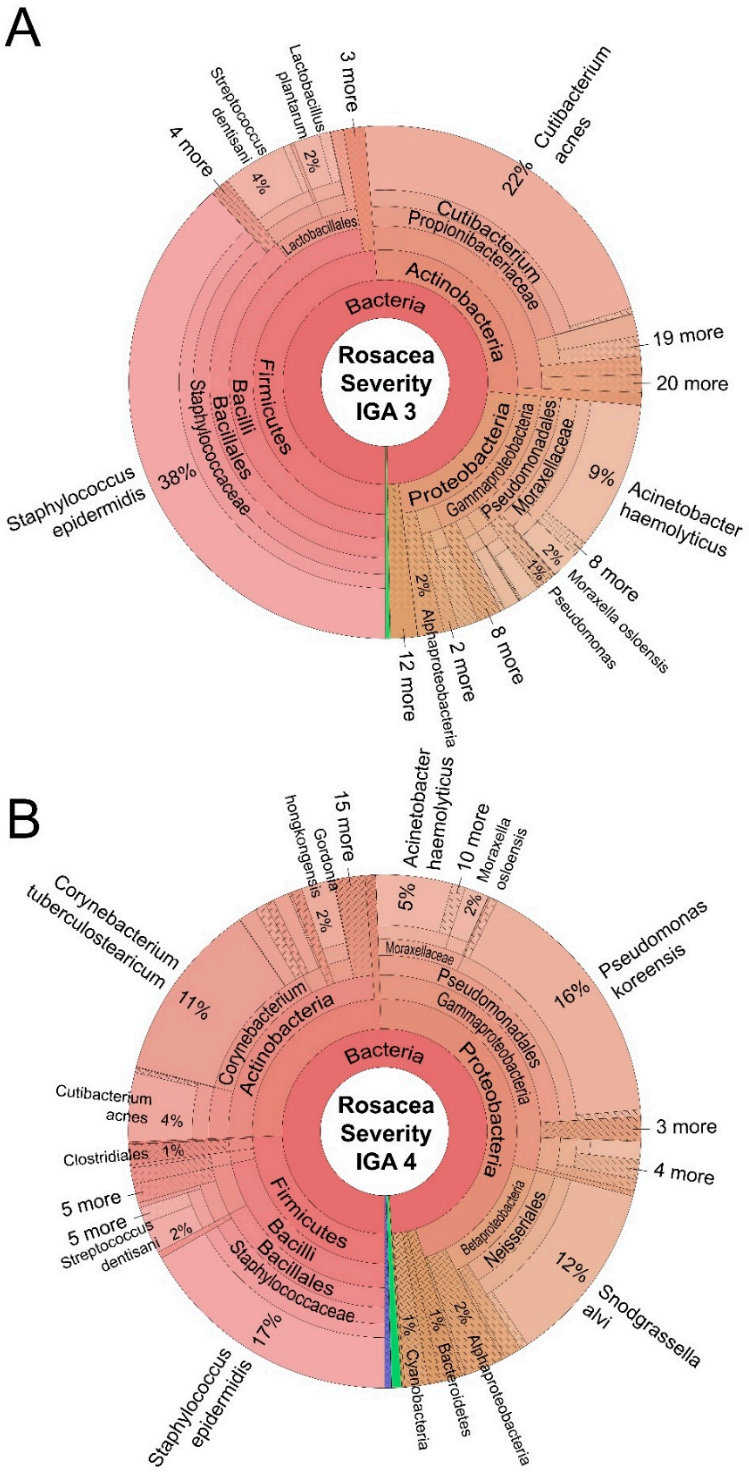

Figure S3. Krona graph on baseline skin microbiota according to rosacea severity. (**A**) IGA 3, and (**B**) IGA 4.
